# Changing role of EMS –analyses of non-conveyed and conveyed patients in Finland

**DOI:** 10.1186/s13049-020-00741-w

**Published:** 2020-05-29

**Authors:** Jani Paulin, Jouni Kurola, Sanna Salanterä, Hans Moen, Nischal Guragain, Mari Koivisto, Niina Käyhkö, Venla Aaltonen, Timo Iirola

**Affiliations:** 1 FinnHEMS Research and Development Unit, FinnHEMS Ltd, Vantaa, Finland; 2 University of Turku (Doctoral Programme in Clinical Research (DPCR) / Medicine), Turku, Finland; 3 Turku University of Applied Sciences, Turku, Finland; 4 Centre for Prehospital Emergency Care, Kuopio University Hospital and University of Eastern Finland, Kuopio, Finland; 5 Department of Nursing Science, University of Turku and Turku University Hospital, Turku, Finland; 6 Department of Future Technologies, University of Turku, Turku, Finland; 7 Department of Biostatistics, University of Turku, Turku, Finland; 8 Department of Geography and Geology, University of Turku, Turku, Finland; 9 Emergency Medical Services, Turku University Hospital and University of Turku, Turku, Finland

**Keywords:** Emergency medical services [MeSH], Non-conveyance, Conveyance

## Abstract

**Background:**

Emergency Medical Services (EMS) and Emergency Departments (ED) have seen increasing attendance rates in the last decades. Currently, EMS are increasingly assessing and treating patients without the need to convey patients to health care facility. The aim of this study was to describe and compare the patient case-mix between conveyed and non-conveyed patients and to analyze factors related to non-conveyance decision making.

**Methods:**

This was a prospective study design of EMS patients in Finland, and data was collected between 1st June and 30th November 2018. Adjusted ICPC2-classification was used as the reason for care. NEWS2-points were collected and analyzed both statistically and with a semi-supervised information extraction method. EMS patients’ geographic location and distance to health care facilities were analyzed by urban–rural classification.

**Results:**

Of the EMS patients (40,263), 59.8% were over 65 years of age and 46.0% of the patients had zero NEWS2 points. The most common ICPC2 code was weakness/tiredness, general (A04), as seen in 13.5% of all patients. When comparing patients between the non-conveyance and conveyance group, a total of 35,454 EMS patients met the inclusion criteria and 14,874 patients (42.0%) were not conveyed to health care facilities. According the multivariable logistic regression model, the non-conveyance decision was more likely made by ALS units, when the EMS arrival time was in the evening or night and when the distance to the health care facility was 21-40 km. Furthermore, younger patients, female gender, whether the patient had used alcohol and a rural area were also related to the non-conveyance decision. If the patient’s NEWS2 score increased by one or two points, the likelihood of conveyance increased. When there was less than 1 h to complete a shift, this did not associate with either non-conveyance or conveyance decisions.

**Conclusions:**

The role of EMS might be changing. This warrants to redesign the chain-of-survival in EMS to include not only high-risk patient groups but also non-critical and general acute patients with non-specific reasons for care. Assessment and on-scene treatment without conveyance can be called the “stretched arm of the emergency department”, but should be planned carefully to ensure patient safety.

## Background

Recently, Emergency Medical Services (EMS) and Emergency Departments (ED) have reported increasing attendance rates [1–3]. An ageing population, lack of social support and difficulties to access primary care were found to be examples of associated factors [3]. During this time, EMS encountered patients are increasingly assessed and treated at the scene; thus avoiding unnecessary conveyances to the ED [4]. In a recent review, non-conveyance rates vary between 3.7 and 93.7% [5]. In Finland, previous studies show that approximately 40% of EMS missions do not lead to patient conveyance to a healthcare facility [6, 7]. Globally these rates are, for example, 19,7% in Sweden [8], 12,9% in Denmark [9], 37.5% in England [10], 26.2% in Netherlands [11] and 15.5% in Australia [12].

The non-conveyance decision-making process seems to be complex [5] and is influenced by several factors from both the EMS staff and patient perspectives [13]. Non-conveyance is part of the EMS process in all types of EMS systems all over the world with patients of all ages, both men and women [5]. The likelihood of non-conveyance is increased as a result of urgency of mission*,* the time of day, a longer distance to a healthcare facility [7], the EMS’ higher educational level [13], the patients’ younger age and a rural area. In contrast, the likelihood of conveyance is increased due to urban areas and patients’ older age [11].

Assessment and triaging of the patient are key elements of an EMS mission [4]. Under-triaging may endanger patients’ safety while over-triaging leads to waste of limited resources [14]. The National Early Warning Score (NEWS2) is a widely adopted simple scoring system developed by the Royal College of Physicians [15]. NEWS2 is used to score deranged physiological parameters like breathing rate, pulse and risk of deterioration [16]. The Royal College of Physicians recommends the use of NEWS2 in prehospital triage [15] and it has been considered a useful triage tool in the prehospital setting [17–19], although the evidence has been questioned [16, 20]. It has to be born in mind that NEWS2 scores physiological parameters only and does not describe the reason for care or degree of disability, which also may influence the conveyance decision.

The main reasons for EMS care vary between non-conveyance and conveyance patients [11]. Internationally, the use of the International Classification of Diseases (ICD10), which indicates the diagnosis of patients, is used, but it has not been developed for EMS. The International Classification of Primary Care (ICPC) was developed for classification of the patient’s reason for an encounter in primary care [21, 22]. ICPC2 is based mainly on symptoms and signs and therefore could be used to also describe the main reason for care in EMS [23].

There is a paucity of evidence available on non-conveyed and conveyed patients, which is why a recent review recommended further insight into the characteristics of the non-conveyance population and comparison between conveyed and non-conveyed patients, including the reasons for care and vital functions [5].

The aim of this study is 1) to describe and compare the patient case-mix between non-conveyed and conveyed patients in EMS and 2) analyze factors related to non-conveyance decision making.

## Methods

### Design

This was a prospective study design.

### EMS in Finland

A national dispatch authority operates with six regional emergency medical communication centers (EMCC) with a common and linked data management system. Incoming calls related to medical incidents are assessed in accordance with a criteria-based and nationally standardized dispatch protocol, which is formulated by national expert panel together with EMCC and the prehospital centers of the five university hospitals. A dispatcher’s training takes 18 months, and the EMCC personnel are usually not health care professionals. After triage, four categories of urgency (A, B, C and D) are used for EMS, where A refers to a life-threatening mission, B to an unknown but potentially high-risk mission, C to an urgent but not life-threatening mission and D to a non-urgent but acute situation.

In Finland, EMS is organized by 21 hospital districts and is a part of specialized healthcare. The structure of EMS is based on a four-tiered system including First Responders (FR), Basic Life Support (BLS) units, Advanced Life Support (ALS) units, on-duty Medical Supervisor units and physician manned units (HEMS and grounds units). The personnel in a BLS unit vary, and consist of firefighters, Emergency medical technicians (EMT) and/or practical or registered nurses. ALS units are the most common, and they are manned by at least one paramedic-nurse with a bachelor level degree requiring 4 years of education or a registered nurse with 1 year of additional education in prehospital emergency care. EMS units mainly operate 24/7. On-scene triaging of a patient is based on national and/or regional treatment protocols and the legal basis is described in the National Health Care Act. After triaging and treatment, units are allowed to make the non-conveyance decision based either on the standing orders or by consulting an EMS physician or doctor in primary care.

In the study area, there are central regional hospitals and municipal healthcare centers or other primary care units, where the EMS can convey patients depending on the urgency and need of the patient. In addition, with some cases it is possible to convey patients to university hospitals located in other areas. For clarity, we use the term healthcare facilities here to refer to all of the above.

### Data collection

**Fig. 1:**
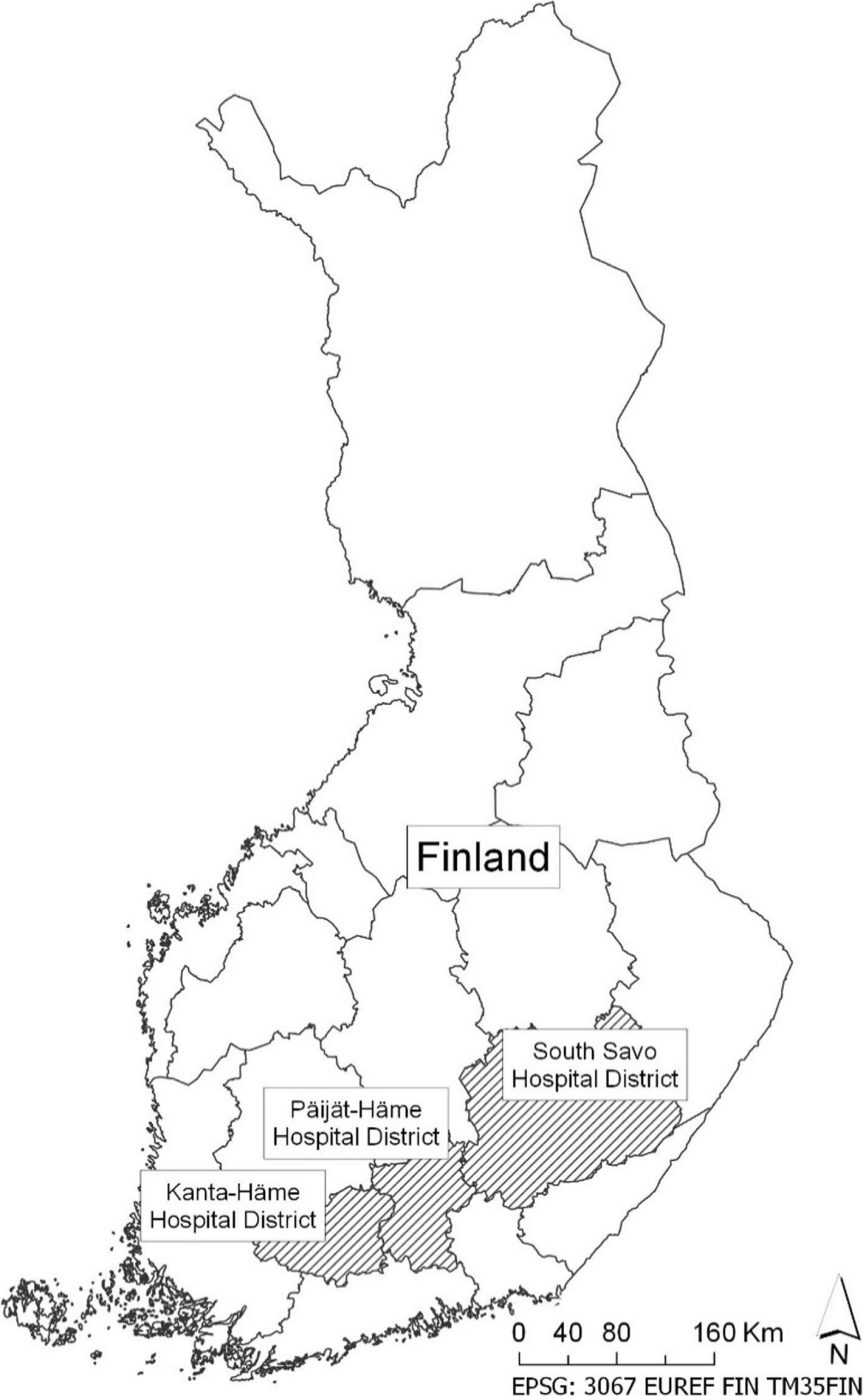
Study areas

The data were collected between 1st June and 30th November 2018 from different data systems of the EMS and hospital patient records in the hospital districts of Etelä-Savo, Kanta-Häme and Päijät-Häme in Finland (Fig. 1). The study area consists of both urban and rural areas, comprising 32 municipalities and a total of 482,805 inhabitants, which is 8.8% of the Finnish population. The average density of population is 26.1 inhabitants per square kilometre.

In the study area, all EMS units were able to use an electronic patient reporting system. There were two different systems in use (Merlot Medi, CGI Suomi Oy, Finland, and Codea, Codea Oy, Finland); data from these EMS databases were combined for further analyses.

The adjusted ICPC2 classification for the EMS reason for care was taken into use; hence the EMS’ electronic patient reporting systems were updated accordingly. The ICPC2 code list used was created by the Nordic Collaboration (Benchmarking) Group for EMS [23]. This list includes around one hundred ICPC2 codes, and this list was proposed to be used in Scandinavian EMS. In this study, the EMS personnel were trained in the use of the codes before the study period.

NEWS2 scores were calculated for the first values that were measured. If there were missing values, they were decoded as normal. The cut-offs for exclusion were as follows: respiration rate < 4/min or > 70/min, oxygen saturation < 50% or > 100%, systolic blood pressure < 40 mmHg or > 280 mmHg, pulse < 20/min, and temperature < 25 °C or > 45 °C. Level of consciousness was assessed by the Glasgow Coma Scale (GCS) in the electronic patient reporting systems. GCS was converted to the ACVPU scale (GCS 15 = Alert (A), GCS 14–3 = CVPU: confusion (C), verbal (V), pain (P), unresponsive (U), similarly as two other studies before [17, 24].

The NEWS2 score calculation requires information about whether supplemental oxygen has been used and if the patient has hypercapnic respiratory failure (usually due to chronic obstructive pulmonary disease, COPD) [15]. We applied a semi-supervised information extraction method to detect and extract relevant mentions from the free-text fields. First the word2vec toolkit [25] was used to train two semantic word space models in an unsupervised manner: one model was trained on a corpus of hospital clinical text (0.9 million physician and nursing notes), and the other was trained on the free text from the EMS data. Next, with a list of keywords related to oxygen administration, hypercapnic respiratory failure and COPD as the starting point (provided by domain experts), we queried the semantic models to extract words with similar meanings. The extracted keyword candidates were then analyzed by the domain experts to notice common synonyms and misspelled variants. This approach is comparable to the interactive rapid vocabulary exploration used in a recent study [26]. With the revised list, we searched and labeled all free-text fields from the EMS missions based on occurrences of these keywords. If the patient had COPD, the SpO2 was analyzed by scale 2 [15].

**Fig. 2:**
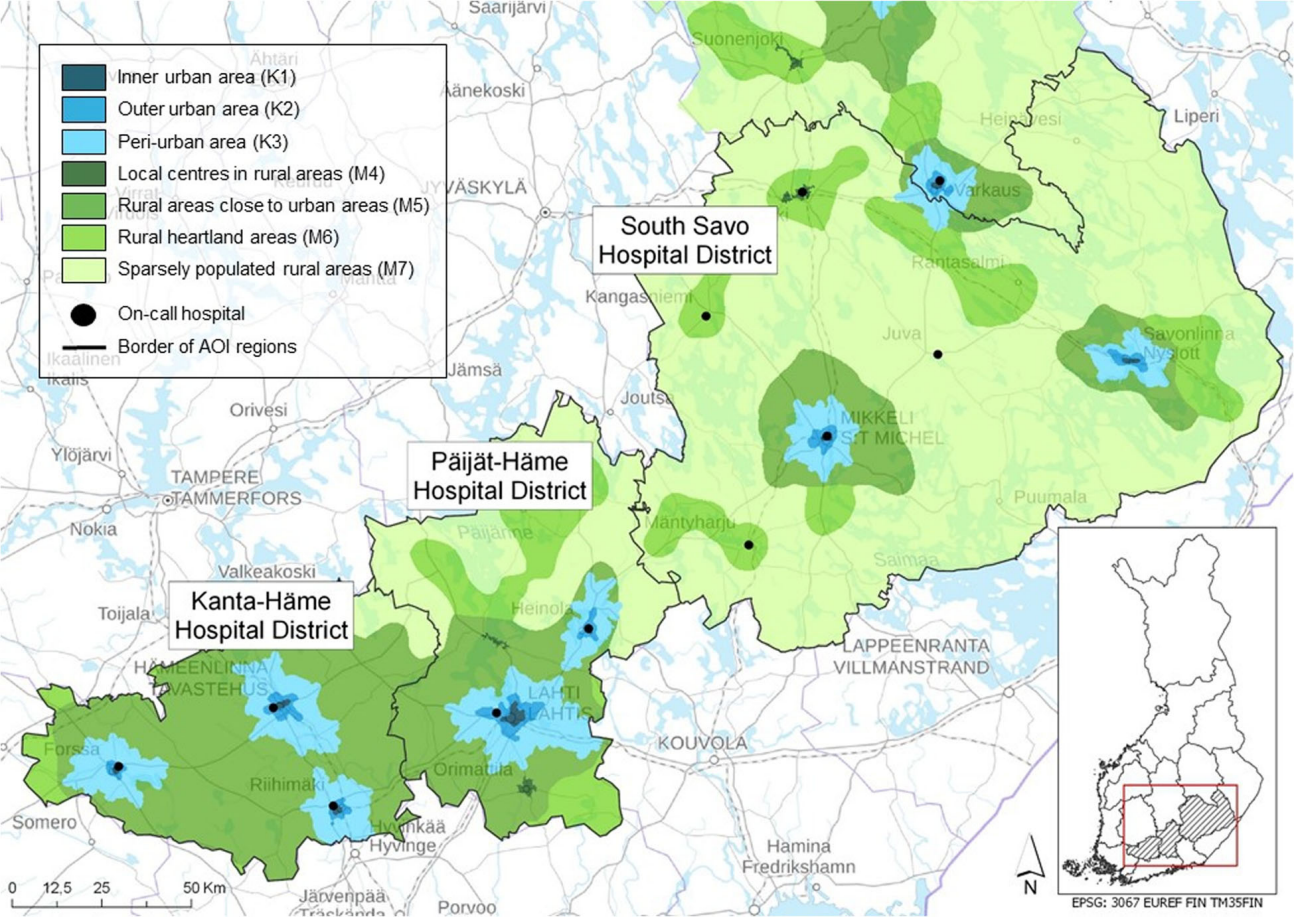
Urban–rural classification [25]

EMS personnel measured the influence of alcohol by a breathalyzer test or clinically. These cases we coded as yes or no into the analyses. The urban-rural classification was analyzed according to the Finnish Environment Institute (SYKE) classification (Fig. 2) [27]. For further statistical analysis, a Spatial Network Analysis was executed for every EMS patient case observation [28, 29]. With the Spatial Network Analysis, the fastest route from every EMS patient mission location to the nearest (non-conveyed patients) or realized (conveyed patients) healthcare facility was calculated. The opening hours of the healthcare facilities were considered in the analysis.

In order to achieve this, three data sets were used when performing the analysis: 1) EMS missions with coordinates, time stamp and other additional information, 2) healthcare facilities with coordinates and opening hours and 3) Digiroad (CC BY 4.0), the national road and street database [30]. All datasets were quality-assessed by their locations and their attribute information and modified, if necessary.

The fastest route from the EMS mission location to the nearest healthcare facility was computed by using the Closest Facility method [31]. The Finnish national road and street database Digiroad (CC BY 4.0) was used as the Network Dataset of the analysis and speed limits of the road network were defined as the cost attribute [30]. Hierarchy and restriction attributes were also defined so that the routing prefers larger main roads, and avoids pedestrian lanes and small forest roads completely.

### Data analyses

Categorical variables were characterized using frequencies and percentages and continuous variables were characterized by using medians and IQR (interquartile range), because variables were not normally distributed. Differences between non-conveyed and conveyance groups were tested using Chi-Square test (categorical variables) or with Mann-Whitney U-test (continuous variables). Univariate associations between non-conveyed and conveyed patients and study variables were studied using logistic regression analysis. Multiple logistic regression analysis included variables that were clinically and statistically significant in univariate analysis, and the expected rural-urban area, because it partly measures the same thing as distance to the nearest healthcare facilities. NEWS2 points were analyzed separately, since it is suitable only for patients over 16 years of age. Results were presented with odds ratios together with 95% confidence intervals and *p*-values. Statistical analyses were carried out using SAS for Windows version 9.4 (SAS Institute Inc., Cary, NC, USA), and *p* values < 0.05 were considered to be statistically significant. The age groups in this study were based on the Finnish national classification provided by Statistics Finland. Distance to health care facilities was classified for the analysis purpose.

## Results

**Fig. 3:**
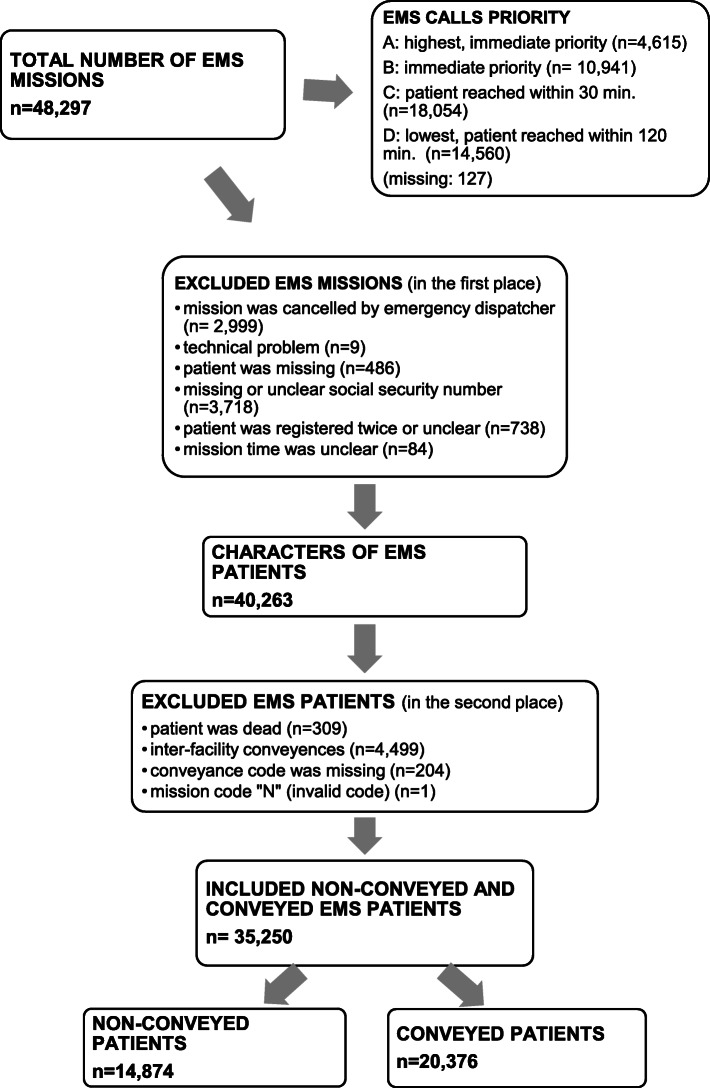
Flow chart

**Fig. 4:**
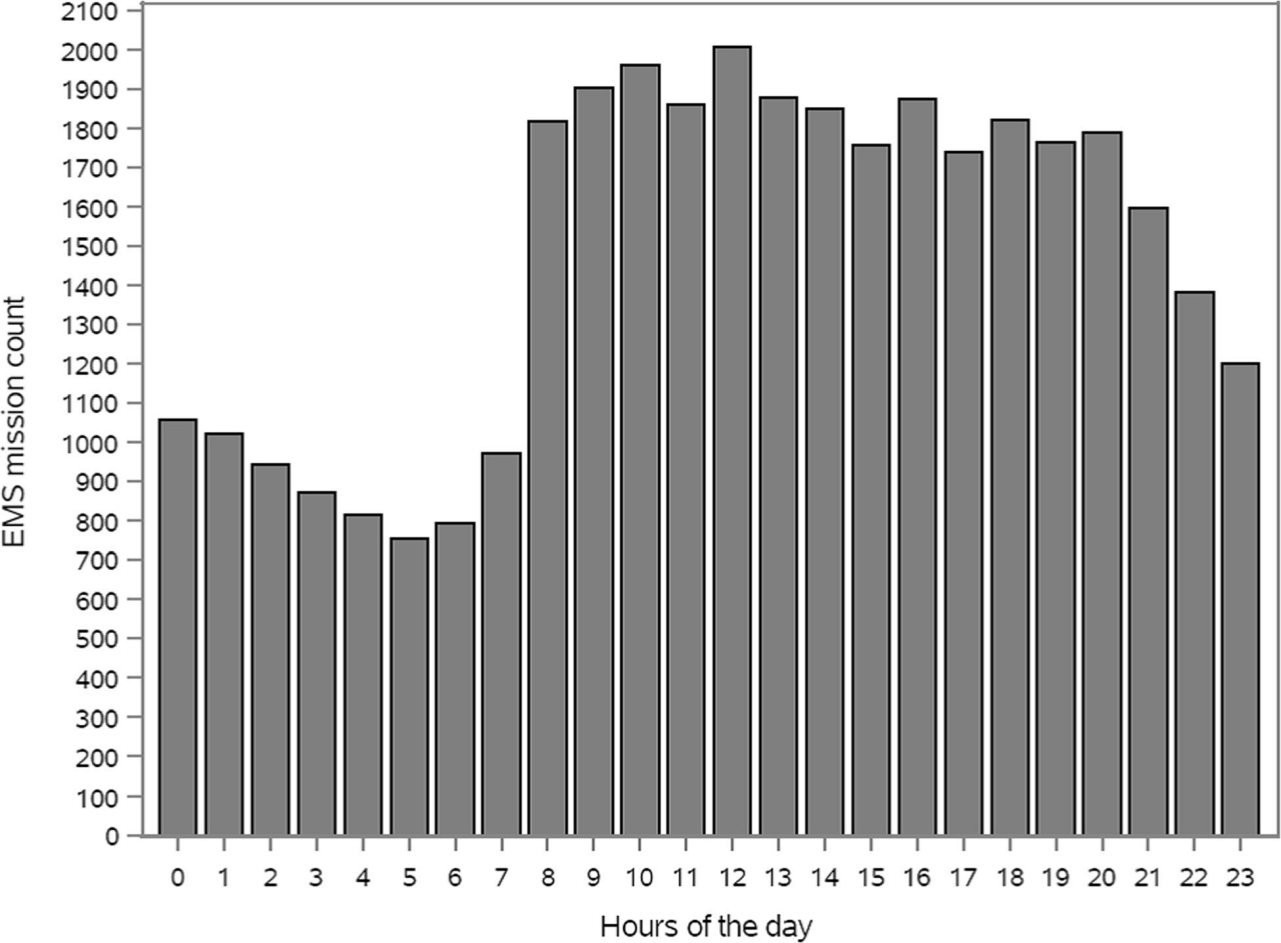
EMS missions per hours

A total of 48,297 EMS missions were identified in the six-month study period. Overall, 40,263 EMS missions were included to describe the characteristics of these missions (Figs. 3 and 4, Table 1). Of the EMS patients, 3.1% were 14 years of age or under, 37.1% were aged between 15 and 64, 40.6% were aged between 64 and 84, and 19.2% were over the age of 85. The median age of the patients was 71 (IQR 51–82) and 51.6% were females. During the six-month study period, 18,449 of the 25,738 patients (71.7%) had one contact with EMS, 6971 patients (27.1%) had 2–6 contacts, and 318 patients (1.2%) had at least seven contacts. The median was one mission and the maximum 86 missions (IQR 1–2). The calculated median of NEWS2 score was 1 point (IQR 0–2), 46.0% of the patients had zero NEWS2 points. According to EMS documentation, 10.0% of the EMS patients were under the influence of alcohol. Table 2 provides an overview of the use of ICPC2 codes.

**Table 1:** Characteristics of EMS missions (40,263)

	Missing	n	%
Mission priority	6		
A		2355	5.9
B		9344	23.2
C		16,019	39.8
D		12,539	31.1
EMS units			
ALS		31,238	77.6
BLS		8491	21.1
Community Paramedic		509	1.3
Field Supervisor		25	0.1
Doctor at scene		265	0.7
Doctor consulted by phone		9670	24.0
Weekday			
Monday		5.687	14.1
Tuesday		5483	13.6
Wednesday		5582	13.9
Thursday		5584	13.9
Friday		6200	15.4
Saturday		6172	15.3
Sunday		5555	13.8
EMS arrival time	47		
08:00–16.00		17,958	44.7
16:00–00:00		14,795	36.8
00:00–08:00		7463	18.6
Urban–rural classification	5733		
Urban area		22,175	64.2
Inner urban area		9230	26.7
Outer urban area		8265	23.9
Peri-urban area		4680	13.6
Rural area		12,355	35.8
Local centres in rural area		1823	5.3
Rural areas close to urban areas		4253	12.3
Rural heartland area		3777	10.9
Sparsely populated rural areas		2502	7.3
Distance to nearest health care facility	5741		
< 5 km		10,969	31.8
5-20 km		11,897	34.5
21-40 km		7250	21.0
> 40 km		4406	12.8
Median distance 8 km, IQR 3.2–25.5			

Mission duration: median 72 min., IQR 51–103

**Table 2:** ICPC2 codes

ICPC2 –codes (*n* = 37,575, missing 2688)			
**General**		n	%
A01	Pain general	566	1.5
A03	Fever	1269	3.4
A04	Weakness/tiredness, general	5060	13.5
A06	Fainting/syncope	708	1.9
A07	Unconsciousness	248	0.7
A78	Suspected sepsis	432	1.2
A80	Major trauma	852	2.3
A81	Multiple trauma	153	0.4
A84	Drug overdose	286	0.8
A85	Side effect of medicine	74	0.2
A86	Toxic effect non-medicinal substance	253	0.7
A87	Complication of surgical procedure	100	0.3
A88.1	Drowning	16	0.04
A88.2	Hypothermia	44	0.1
A88.3	Hyperthermia	16	0.04
A88.4	Pressure related disease	1	0.00
A92	Allergy/allergic reaction NOS	307	0.8
A95	SIDS	4	0.01
A96	Death	167	0.4
A97	No disease	1635	4.4
**Gastrointestinal**		n	%
D01	Acute abdomen	1663	4.4
D09	Nausea	243	0.7
D10	Vomiting	283	0.8
D11	Diarrhoea	178	0.5
D14	Haematemesis/vomiting blood	81	0.2
D15	Melaena	34	0.1
D16	Rectal bleeding	144	0.4
**Eye**		n	%
F01	Eye pain	7	0.02
F29	Eye symptom/complaint other	78	0.2
F76	Foreign body in eye	3	0.01
F79	Injury eye other	27	0.1
**Ear**		n	%
H01	Ear pain	5	0.01
H29	Ear symptom/complaint other	15	0.04
H76	Foreign body in ear	1	0.00
H79	Ear injury other	6	0.02
**Cardiac and circulation**			
A11	Chest pain	985	2.6
K29	Cardiovascular symptom/complaint other	188	0.5
K74	Iscaemic chest pain	860	2.3
K75	Acute myocardial infarction	129	0.3
K77	Acute heart failure	276	0.7
K78	Atrial fibrillation/flutter	611	1.6
K79	Paroxysmal tachycardia	87	0.2
K80	Other cardiac arrhythmia	804	2.1
K85	High blood pressure	263	0.7
K89	Transient cerebral ischaemia (TIA)	258	0.7
K90	Stroke	866	2.3
K93	Pulmonary embolism	34	0.1
K98	Cardiac arrest	126	0.3
K99	Suspected of aortic aneurysm	23	0.1
**Musculoskeletal**			
L01	Neck symptom/complaint	193	0.5
L02	Back symptom/complaint	983	2.6
L04	Chest symptom/complaint	224	0.6
L05	Flank/axilla symptom/complaint	173	0.5
L08	Shoulder symptom/complaint	248	0.7
L09	Arm symptom/complaint	120	0.3
L10	Elbow symptom/complaint	48	0.1
L11	Wrist symptom/complaint	73	0.2
L12	Hand/finger symptom/complaint	145	0.4
L13	Hip symptom/complaint	681	1.8
L14	Leg/thigh symptom/complaint	222	0.6
L15	Knee symptom/complaint	311	0.8
L16	Ankle symptom/complaint	148	0.4
L17	Foot/toe symptom/complaint	443	1.2
L29	Musculoskeletal symptom/complaint other	158	0.4
**Acute injury related**			
L76	Fracture	159	0.4
L79	Sprain/strain of joint NOS	10	0.03
L80	Dislocation/subluxation	33	01
L81	Injury musculoskeletal NOS	184	0.5
**Nervous system**			
N01	Headache	496	1.3
N07	Convulsion/seizure	832	2.2
N17	Vertigo/dizziness	1033	2.8
N80	Head injury	994	2.7
N81	Spinal trauma	41	0.1
**Psychiatric**			
P16	Acute alcohol abuse	1597	4.3
P19	Drug abuse (not medicinal drugs)	121	0.3
P29	Psychological symptom / complaint other	1660	4.4
P77	Suicide/suicide attempt	86	0.2
P98	Psychosis	198	0.5
**Respiratory**			
R01	Pain respiratory system	39	0.1
R02	Shortness of breath / dyspnoea	1607	4.3
R05	Cough	41	0.1
R06	Nose bleed/epistaxis	242	0.6
R29	Respiratory symptom / complaint other	284	0.8
R77	Laryngitis	21	0.1
R83	Respiratory infection other	221	0.6
R87	Foreign body nose / larynx	68	0.2
R95	COPD	74	0.2
R96	Asthma	41	0.1
R98	Hyperventilation syndrome	107	0.3
**Skin**		n	%
S12	Insect bite/sting	48	0.1
S13	Animal/human bite	18	0.1
S14	Burn/scald	59	0.2
S15	Foreign body in skin	4	0.01
S16	Bruise/contusion	119	0.3
S18	Laceration/cut	1024	2.7
S29	Skin symptom/complaint other	47	0.1
**Endocrinology**		n	%
T11	Dehydration	86	0.2
T87	Hypoglycaemia	174	0.5
A91	Hyperglycemia	164	0.4
**Urinary**		n	%
U08	Urinary retention	93	0.3
U29	Urinary symptom/complaint other	351	0.9
**Female genital**		n	%
X29	Genital symptom/complaint female other	37	0.1
X82	Injury genital female	2	0.01
**Male genital**		n	%
Y29	Genital symptom/complaint male other	26	0.1
Y80	Injury male genital	9	0.02
**Social**		n	%
Z25	Assault/harmful event problem	198	0.5
Z29	Social problem NOS	193	0.5
**Pregnancy and childbirth**		n	%
W03	Antepartum bleeding	23	0.1
W29	Pregnancy symptom/complaint other	43	0.1
W90	Uncomplicated delivery livebirth	19	0.1
W92	Complicated delivery livebirth	9	0.02
W93	Complicated labour/delivery stillbirth	1	0.00

When comparing patients between the non-conveyance and conveyance group, a total of 35,454 EMS patients met the inclusion criteria (Fig. 3). Of the total patients, 14,874 (42.0%) were treated at scene and 20,376 patients (58.0%) were conveyed to healthcare facilities. Non-conveyance decisions were based on different causes (Table 3).

**Table 3:** Reasons for non-conveyance (*n* = 14,874)

	n (*n* = 14,874)	% (100%)
Non-conveyed EMS patients were treated at scene or there was no need for conveyance.	10,713	72.0
Patients were taken to healthcare facilities in their own or relatives’ car or by taxi, for example.	3013	20.3
Patients refused conveyance.	736	5.0
Patients were handed over to the police.	306	2.1
Patients received other help, such as homecare.	106	0.7

NEWS2 scores of these groups are presented in Table 4. The most frequent ICPC2 codes in the non-conveyance group were weakness/tiredness, general (A04) (*n* = 1929), no disease (A97) (*n* = 1412) and acute alcohol abuse (P16) (*n* = 966), and in the conveyance group, weakness/tiredness, general (A04) (*n* = 2614), psychological symptom/complaint other (P29) (*n* = 1049) and shortness of breath/dyspnoea (R02) (*n* = 987). The numbers of these ICPC2 codes are different from those in Table 2 due the exclusion of deceased and inter-facility conveyed patients. The time spent by EMS units at the scene was longer with non-conveyed patients (median 28 min., IQR 20–37, missing 70) than with conveyed patients (median 24 min., IQR 17–32, missing 31) (p = < 0.001). When patients were treated and left at the scene, doctors were consulted in 39% of the cases; if patients were conveyed, the corresponding figure was 18% (*p* = 0.001).

**Table 4:** NEWS2 score (age over 16 years)

		All patients (*n* = 38,788)	Non-conveyed patients (*n* = 13,723) (missing 134)	Conveyed patients (*n* = 19,727) (missing 134)
**NEWS2 score**	**Clinical risk**	**n (%)**	**n (%)**	**n (%)**
Aggregate score 0–4	Low	31,397 (81.0)	13,160 (90.8)	18,055 (74.9)
Red score; Score of 3 in any individual parameter	Low–medium	4049 (10.4)	983 (6.8%)	3058 (12.7)
Aggregate score 5–6	Medium	2076 (5.4)	265 (1.8)	1805 (7.5)
Aggregate score 7 or more	High	1256 (3.2)	79 (0.6)	1176 (4.9)
Median + IQR		median 1, IQR 0–2	median 0, IQR 0–1	median 1, IQR 0–3

The multivariable logistic regression model of non-conveyed patients is shown in Table 5. Based on our data, non-conveyance was associated with mission priority D (OR 1.629, 95% CI: 1.527–1.736) and C (OR 1.520, 95% CI: 1.436–1.608) more often than B. A non-conveyance decision was more likely made by ALS units (OR 1.240, 95% CI: 1.170–1.315), in EMS arrival times between 16:00–00.00 (OR 1.310, 95% CI: 1.245–1.379) and 00.00–8:00 (OR 1.711, 95% CI: 1.610–1.818) than in the daytime, and was more likely at night than in the evening (OR 1.306, 95% CI: 1.230–1.387). The distance 21–40 km to healthcare facilities was also related to non-conveyance (21–40 km vs 5 km OR 1.147, 95% CI: 1.077–1.221, 21–40 km vs 5–20 km OR 1.233, 95% CI: 1.159–1.311, 21–40 km vs > 40 km OR 1.263, 95% CI: 1.167–1.367. Non-conveyance decision was also more likely made on patients with younger age (15–64 vs 65–84 OR 1.206, 95% CI: 1.145–1.271), female gender (OR 1.128, 95% CI: 1.077–1.181) and if the patient had used alcohol (OR 1.473, 95% CI: 1.370–1.585). All differences were significant (*p* < 0.001). Odd ratios changed in multivariable analyses, but all odd ratios remained significant compared to univariate analyses.

**Table 5:** Multivariate logistic regression model (n = 14,874)

	Missing	Univariate			Multivariate		
		OR	95% CI	*р*	OR	95% CI	*р*
Mission priority	210						
C vs B		1.504	1.424–1.588	< 0.001	1.520	1.436–1.608	< 0.001
D vs B		1.548	1.459–1.643	< 0.001	1.629	1.527–1.736	< 0.001
B vs A		1.869	1.672–2.088	< 0.001	1.916	1.708–2.150	< 0.001
C vs A		2.810	2.524–3.127	< 0.001	2.912	2.605–3.256	< 0.001
D vs A		2.893	2.593–3.228	< 0.001	3.121	2.780–3.503	< 0.001
EMS units ALS vs BLS	142	1.187	1.124–1.253	< 0.001	1.240	1.170–1.315	< 0.001
EMS arrival time	231						
16:00–00.00 vs 08:00–16.00		1.466	1.397–1.538	< 0.001	1.310	1.245–1.379	< 0.001
00:00–08:00 vs 08:00–16:00		1.835	1.733–1.943	< 0.001	1.711	1.610–1.818	< 0.001
00:00–08:00 vs 16:00–00:00		1.252	1.181–1.326	< 0.001	1.306	1.230–1.387	< 0.001
Distance to nearest health care facilities	1492						
21-40 km vs < 5 km		1.188	1.118–1.261	< 0.001	1.147	1.077–1.221	< 0.001
21-40 km vs 5-20 km		1.238	1.167–1.314	< 0.001	1.233	1.159–1.311	< 0.001
21-40 km vs > 40 km		1.317	1.219–1.421	< 0.001	1.263	1.167–1.367	< 0.001
Age	204						
< 15 vs 15–64		1.392	1.232–1.574	< 0.001	1.723	1.515–1.960	< 0.001
< 15 vs 65–85		1.727	1.528–1.952	< 0.001	2.078	1.828–2.363	< 0.001
< 15 vs > 85		2.027	1.784–2.303	< 0.001	2.419	2.114–2.768	< 0.001
15–64 vs 65–84		1.241	1.183–1.301	< 0.001	1.206	1.145–1.271	< 0.001
15–64 vs > 85		1.456	1.371–1.546	< 0.001	1.404	1.313–1.501	< 0.001
65–84 vs > 85		1.173	1.105–1.246	< 0.001	1.164	1.092–1.240	< 0.001
Gender female vs male	204	1.049	1.006–1.094	< 0,027	1.128	1.077–1.181	< 0.001
Alcohol		1.708	1.599–1.826	< 0.001	1.473	1.370–1.585	< 0.001

Furthermore, the univariate analyses show that a rural area (OR 1.465, 95% CI: 1.401–1.533) and a low NEWS2 score (0–4 vs 5–6 OR 5.222, 95% CI: 4.555–5.987, 0–4 vs score of 3 in any individual parameter OR 2.713, 95% CI: 2.501–2.944) also increased the likelihood of non-conveyance. There was no evidence that mission priority D was more likely treated at the scene than priority C. (*p* < 0.001, OR 1.014, 95% CI: 0.963–1.068). Our data also indicates that if there was less than an hour to complete a shift, it did not relate to the non-conveyance decision (*p* = 0.491).

In contrast, our multivariable logistic regression analyses (Table 6) indicate that, for example, conveyed patients were older (65–84 vs 15–64 OR 1.282, 95% CI: 1.218–1.349) and more likely male (OR 1.095, 95% CI: 1.046–1.146). The univariate analyses show that an urban area (OR 1.465, 95% CI: 1.401–1.533) and whether the patient’s NEWS2 score increases by one point (OR 1.377, 95% CI: 1.357–1.398) or by two points (OR 1.897, 95% CI: 1.842–1.954), increased the likelihood of conveyance as well. All differences were significant (p < 0.001). The results also show that if there was less than an hour to complete a shift, it did not relate to the conveyance decision, either (p = 0.491).

**Table 6:** Multivariate logistic regression analyses of conveyed patients (*n* = 20,376)

	Missing	Univariate			Multivariate		
		OR	95% CI	*р*	OR	95% CI	*р*
Mission priority	210						
A vs C		2.810	2.524–3.127	< 0.001	2.987	2.672–3.340	< 0.001
B vs C		1.504	1.424–1.588	< 0.001	1.540	1.455–1.629	< 0.001
A vs D		2.893	2.593–3.228	< 0.001	3.193	2.845–3.584	< 0.001
B vs D		1.548	1.459–1.643	< 0.001	1.646	1.544–1.755	< 0.001
EMS units ALS vs BLS	142	1.187	1.124–1.253	< 0.001	1.231	1.161–1.305	< 0.001
EMS arrival time	231						
08:00–16.00 vs 16:00–00:00		1.466	1.397–1.538	< 0.001	1.345	1.278–1.415	< 0.001
08:00–16.00 vs 00:00–08:00		1.835	1.733–1.943	< 0.001	1.771	1.667–1.881	< 0.001
16:00–00:00 vs 00:00–08:00		1.252	1.181–1.326	< 0.001	1.317	1.240–1.398	< 0.001
Distance to nearest health care facilities	1492						
< 5 km vs 21-40 km		1.188	1.118–1.261	< 0.001	1.125	1.057–1.197	< 0.001
5-20 km vs 21-40 km		1.238	1.167–1.314	< 0.001	1.233	1.160–1.311	< 0.001
> 40 km vs 21-40 km		1.317	1.219–1.421	< 0.001	1.258	1.163–1.362	< 0.001
Age	204						
15–64 vs < 15		1.392	1.232–1.574	< 0.001	1.596	1.404–1.813	< 0.001
65–85 vs < 15		1.727	1.528–1.952	< 0.001	2.045	1.799–2.326	< 0.001
> 85 vs < 15		2.027	1.784–2.303	< 0.001	2.423	2.118–2.773	< 0.001
65–84 vs 15–64		1.241	1.183–1.301	< 0.001	1.282	1.218–1.349	< 0.001
> 85 vs 65–84		1.173	1.105–1.246	< 0.001	1.185	1.112–1.262	< 0.001
Gender female vs male	204	1.049	1.006–1.094	< 0,027	1.095	1.046–1.146	< 0.001

## Discussion

The main findings in this study were; firstly, 42% of EMS patients were non-conveyed to health care facilities. Secondly, NEWS2 points were low, almost half of the patients had zero points. Thirdly, the adjusted ICPC2 was used for the first time in EMS and it showed that the most common reasons for care were general and non-specific complaints.

Our study shows that over 40% of EMS patients were non-conveyed after assessment and care, which demonstrates the changing role of EMS towards more acute mobile healthcare [32]. According to a recent review, non-conveyance rates vary between 3.7 and 93.7% in general populations in EMS [5]. Even though one study has highlighted the fact that non-conveyance rates vary in different areas [33], our findings are similar to two other previous Finnish studies [6, 7].

We found a new, multivariate logistic regression model (Table 5), which indicates, not surprisingly, that rather than mission priority A or B, priorities C and D were related to non-conveyance. Surprisingly, according to the univariate analyses, mission priority D did not predict any increase in non-conveyance than C. On the other hand, the dispatch process in Finland has been questioned especially related to the accuracy of non-life-threatening situations [7], and unnecessary EMS missions arouse debate globally [34]. It seems that the number of missions, which do not require any medical intervention from an EMS unit, is increasing [1]. Although, it’s universally accepted that the dispatch process is designed to recognize life-threatening incidents, it seems that majority of emergency calls are related to non-life threatening incidents which challenges the use of EMS resources. Ideally, high specifity and sensitivity is required in both patient groups. It is also notable that EMCC personnel’s education varies between countries. In Finland, like mentioned before, formally dispatchers are not required to have health care degree [35]. However, more studies are needed.

Our model also indicates that the non-conveyance decision was more likely made by ALS units, which is consistent with a previous study [13]. ALS units’ higher education and competence partly explain these differences in non-conveyance decision making. EMS arrival time in the evening or at night is similar to the findings of a previous study as well [7]. One reason might be the fact that primary care access is better in the daytime. Furthermore, one study found that EMS patients between 5 pm–7 am do not usually require much treatment [36].

Our model demonstrates that younger patients were more likely not to be conveyed than the elderly. Younger patients have previously been related to non-conveyed patients [11] and, for example, one study highlighted that patients under 25 years rarely needed any treatment [36]. On the other hand, a review found that geriatric patient groups were common among these patients and one fourth of the non-conveyed patients were 70 years or older [5]. This is surprising because it is commonly recognized, that elderly patients are a challenging patient group to assess and treat [13, 37]. We found that elderly patients were more likely conveyed to health care facilities. Overall, 10.0% of EMS patients were under the influence of alcohol, which is less than another study found [6]. However, according to our model, alcohol was associated with a non-conveyance decision.

There are some non-patient factors related to non-conveyance decisions. At the end of a work shift, it might be an easy option to convey patients to healthcare facilities [13]. However, our data indicates that if there was less than 1 h to complete a shift, it was not associated with either the non-conveyance or conveyance decision. Thus, it seems that, EMS take into account the patients’ needs, even if a shift is almost complete. Our study indicates that a non-conveyance decision is more time-consuming than a conveyance decision, which is in line with previous studies [13, 38]. There were also more doctors’ consultations for decision making in the non-conveyance group (39%) than in the conveyance group (18%). This is consistent with the other study [13]. The distance 21–40 km to healthcare facilities was associated with non-conveyance decisions compared to shorter or longer distances. EMS care providers might think that conveyance is easy when the distance is short, and in the case of longer distances, there might be too many risks involved with a non-conveyance decision. Previous studies have concluded that there are less non-conveyed patients in rural areas [11, 38]. We found, in the univariate analyses, that rural area increased the likelihood of non-conveyance. One explanation might be that in one area, four out of six healthcare facilities were in the rural area. Moreover, in Finland, the urban-rural classification is entirely different when compared to many other countries because Finland is a very sparsely populated country.

Of the patients, 46.0% had zero NEWS2 points, which is a high percentage compared to previous research [7]. The results show that even if EMS traditionally handle critically ill patients, a notable proportion of EMS patients are in relatively good condition. Abnormal vital signs are common predictors of all subsequent events [12], and a study found that two out of three conveyed patients had one or more abnormal vital functions [11]. A review indicates that patients with a score of NEWS 0 are very unlikely to deteriorate, and patients with high scores (NEWS ≥7) were more likely to deteriorate, but evidence of intermediate scores (1–6) is unclear [16]. We found that if the patient’s NEWS2 score increases by one point or two points, the likelihood of conveyance increased.

To our knowledge, this was the first study where the adjusted ICPC2 classification for emergency care was used. The most common code was weakness/tiredness, general (A04), seen in 13.5% of all patients. This is in concordance from recent finding from emergency departments [39]. Overall, it also seems that traditional high risk patient groups such as “first hour quintet” [40] represent a small minority of all patients encountered by the EMS. According to a recent review, there were a considerable number of patients with a variety of initial complaints and conditions [5]. Not surprisingly, no disease (A97) was a common code in the non-conveyance group, as was acute alcohol abuse (P16), as was found earlier in Finland [6]. Our study showed that patients in the conveyed group were more likely to suffer mental problems (P29), which is the same result as in previous studies [41, 42]. This is surprising, because one study found that psychiatric patients rarely need any treatment [36]. On the other hand, there is evidence that EMS units often lack the skills required to manage these patients [41]. However, another study found a contrary result, i.e. patients with mental health problems were more likely to be treated and left at the scene [11]. Shortness of breath/dyspnoea (R02) was also a common reason for patients in the conveyance group, which was also seen in a previous study [11]. It can be said that the adjusted ICPC2 gave more detailed information concerning patient care and a deeper insight than emergency dispatch codes. ICD10 is designed for diagnostic purposes and its use in EMS can be questioned. Therefore, it seems that the adjusted ICPC2 classification might be feasible option in prehospital emergency care to describe and classify reason for care, but more research is needed.

### Limitations

This study has some limitations. First, although this study had a prospective design, the registries used were not primarily designed for scientific research, which is why we had to exclude a number of patients. For instance, 3718 patients were missing or had an incorrect or unclear social security number. Second, the use of adjusted ICPC2 classification for emergency care was only recently adopted; consequently, its usage was not established despite the training of the EMS personnel. In one study area, due to a human error, ICPC2 codes Z25 and Z29 (social problems) were missing, although those codes were quite rare. We only looked at the main reason for care, even if another ICPC2 code, also chosen by EMS, might have provided more information about the patient. Third, when we analyzed the NEWS2 points, we interpreted the missing values as normal. The final NEWS2 scores obtained may thus be lower. We analyzed the use of oxygen and whether the patient had a COPD with the text mining method (machine learning). Even if we noted spelling mistakes etc., it is possible that we did miss some information. For example, trade names of drugs change constantly. Furthermore, the level of consciousness was assessed by GCS, which we converted to the AVCPU scale. In the ACVPU scale, C means new confusion [15], which is difficult to detect retrospectively, but it does not change this analysis compared to the conversion of GSC to AVPU. Fourth, the EMS units’ non-conveyance decision-making was influenced by several other factors, which were not analyzed in this study. Requirements of the EMS, organizational support, guidelines and human factors differ between study areas. Fifth, it should be noted that in this article we did not assess the outcomes, follow-up care or patient safety of non-conveyed and conveyed patients.

However, despite of these limitations, we feel that the aims of this study are justified. In this prospective study design we collected data from all EMS missions from three areas over six-month period. The dataset is large and a wide range of variables were tested. The characteristics of EMS patients were described, differences between non-conveyed and conveyed patients were shown, and multivariate logistic regression model was found.

## Conclusions

In this study high rate of non-conveyance, non-spesific reasons for EMS care and low NEWS2 points, indicate that the overall role of the EMS in acute health care might be changing. This points out that especially the role of primary health care services might have changed. This warrants to redesign the chain-of-survival in EMS to include not only high-risk and critical patient groups but also non-critical and general acute patients with non-specific reasons for care. Assessment and on-scene treatment without conveyance can be described as the “stretched arm of emergency department” but should be planned carefully and together with emergency departments and primary health care to ensure patient safety.

## Data Availability

The data of this study is not available due to patients’ privacy.

## Abbreviations

EMS: Emergency medical services
NEWS2: National Early Warning Score
